# Medication and nutritional supplement use before and after bariatric surgery: Utilização de medicamentos e suplementos nutricionais antes e após a cirurgia bariátrica

**DOI:** 10.1590/1516-3180.2015.0241030516

**Published:** 2016-10-31

**Authors:** Charline Fernanda Backes, Edyane Lopes, Airton Tetelbom, Isabela Heineck

**Affiliations:** I Master’s Student in the Postgraduate Pharmaceutical Sciences Program, Universidade Federal do Rio Grande do Sul (UFRGS), Porto Alegre, RS, Brazil.; II PhD. Pharmacist, School of Public Health, Health Department of the State of Rio Grande do Sul, Porto Alegre, RS, Brazil.; III MD. Coordinator of the Health Technology Assessment Center, Grupo Hospitalar Conceição; Head Professor of Public Health, Universidade Federal de Ciências da Saúde de Porto Alegre (UFCSPA); Associate Professor of Public Health, Universidade Luterana do Brasil (ULBRA); and Contributing Professor in the Postgraduate Epidemiology Program, Department of Social Medicine, School of Medicine, Universidade Federal do Rio Grande do Sul (UFRGS), Porto Alegre, RS, Brazil.; IV PhD. Associate Professor, Postgraduate Pharmaceutical Sciences Program and Postgraduate Pharmaceutical Services, School of Pharmacy, Universidade Federal do Rio Grande do Sul (UFRGS), Porto Alegre, RS, Brazil.

**Keywords:** Bariatric surgery, Comorbidity, Pharmaceutical preparations, Gastric bypass, Obesity, morbid, Cirurgia bariátrica, Comorbidade, Preparações farmacêuticas, Derivação gástrica, Obesidade mórbida

## Abstract

**CONTEXT AND OBJECTIVE:**

Bariatric surgery has been an effective alternative treatment for morbid obesity and has resulted in decreased mortality, better control over comorbidities and reduced use of drugs. The objective of this study was to analyze the impact of bariatric surgery on medication drug and nutritional supplement use.

**DESIGN AND SETTING:**

Longitudinal study of before-and-after type, on 69 morbidly obese patients in a public hospital in Porto Alegre.

**METHODS:**

Through interviews, the presence of comorbidities and use of drugs with and without prescription were evaluated.

**RESULTS:**

Among the 69 patients interviewed, 85.5% had comorbidities in the preoperative period, with an average of 2.3 (± 1.5) per patient. The main comorbidities reported were hypertension, diabetes and dyslipidemia. 84.1% of the patients were using prescribed drugs in the preoperative period. The mean drug use per patient was 4.8, which decreased to 4.4 after the procedure. The surgery enabled significant reduction in use of most antidiabetic (84%), antilipemic (77%) and antihypertensive drugs (49.5%). On the other hand, there was a significant increase in use of multivitamins and drugs for disorders of the gastrointestinal tract. The dosages of most of the drugs that continued to be prescribed after surgery were decreased, but not significantly.

**CONCLUSION:**

After bariatric surgery, there were increases in the use of vitamins, gastric antisecretory drugs and antianemic drugs. Nevertheless, there was an overall reduction in drug use during this period, caused by suspension of drugs or dose reduction.

## RESUMO

**CONTEXTO E OBJETIVO:**

A cirurgia bariátrica tem sido uma alternativa efetiva de tratamento para a obesidade mórbida, resultando na diminuição da mortalidade, melhor controle das comorbidades e redução no uso de medicamentos. O objetivo deste estudo foi analisar o impacto da cirurgia bariátrica sobre a utilização de medicamentos e suplementos nutricionais.

**TIPO DE ESTUDO E LOCAL:**

Estudo longitudinal do tipo antes e depois com 69 pacientes obesos mórbidos em um hospital público de Porto Alegre.

**MÉTODOS:**

Nas entrevistas, foram avaliados a presença de comorbidades e o uso de medicamentos com e sem prescrição médica.

**RESULTADOS:**

De 69 pacientes entrevistados, 85,5% apresentaram comorbidades no período pré-cirúrgico, média de 2,3 (± 1,5) por paciente. As principais comorbidades relatadas foram hipertensão, diabetes e dislipidemias. 84,1% dos pacientes estavam em uso de medicamentos sob prescrição médica, no período pré-cirúrgico. A média de uso de medicamentos por paciente foi de 4,8, reduzindo para 4,4 após o procedimento. A cirurgia proporcionou diminuição significativa do uso da maioria dos antidiabéticos (84%), antilipêmicos (77%) e anti-hipertensivos (49,5%). Por outro lado, observou-se aumento significativo na utilização de multivitamínicos e medicamentos para desordens do trato gastrointestinal. A maior parte dos medicamentos que continuaram sendo prescritos após a cirurgia teve sua dose reduzida, no entanto, esta redução não foi significativa.

**CONCLUSÃO:**

Após a cirurgia bariátrica, observou-se aumento na utilização de vitaminas, antisecretores gástricos e antianêmicos. No entanto, de forma geral, houve redução na utilização de medicamentos neste período, ocasionada pela suspensão de medicamentos ou redução de doses.

## INTRODUCTION

Chronic non-communicable diseases are one of the biggest public health issues today.^1^ Obesity stands out in this regard and has been officially acknowledged by the World Health Organization (WHO) as a chronic disease.^2^

The number of obese individuals has been increasing worldwide and has reached an average of 30% of the adult population in some countries.^3^ In Brazil, poor dietary habits have been reflected in the population’s health and in increasing prevalence of overweight. More than half of all Brazilians (51%) are now overweight, and 17% of these individuals are obese.^1^

Fighting this rapid growth is one of the biggest challenges for world health, given that obesity is frequently associated with a vast array of comorbidities, such as systemic arterial hypertension, type 2 diabetes mellitus, dyslipidemia, obstructive sleep apnea, cardiovascular diseases and some types of cancer.^4^^,^^5^^,^^6^^,^^7^^,^^8^ Most patients with high blood pressure are overweight,^9^ and hypertension is six times more frequent among obese individuals than among those with normal weight.^10^

The alternatives to clinic treatment for reducing weight among patients with morbid obesity are limited, and the long-term outcomes are relatively inefficient.^11^ Bariatric surgery seems to be a viable option for treating morbid obesity, since it has been shown to be effective in maintaining the weight loss. A significant improvement in comorbidities and even their regression may be observed in most patients who undergo this surgical procedure for weight loss.^10^ This surgical procedure has been shown to enable control over glucose levels, even leading to remission of diabetes.^12^ The quick and sustained improvement in glucose homeostasis that this procedure provides has made it the gold-standard metabolic procedure and treatment for diabetic patients with morbid obesity.^13^ Comorbidity reductions are reflected in diminished drug use during the postoperative period, and discontinuation of drug therapy for some diseases, thus resulting in reduced expenditure on drugs and other healthcare services.^10^^,^^14^^,^^15^^,^^16^^,^^17^^,^^18^

In addition, bariatric surgery extends survival, decreases occurrences of cardiovascular events and is also associated with greater reduction of mortality due to cardiovascular diseases, myocardial infarction, stroke, diabetes and cancer, in comparison with obese individuals who do not undergo this surgery.^10^^,^^19^^,^^20^ The reduction in the risk of myocardial infarction, stroke and adverse cardiovascular events is approximately 50% after this surgery, in comparison with individuals who did not undergo the procedure.^19^

If, on the one hand, bariatric surgery reduces the need for medication to treat comorbidities; on the other hand, the restrictive and disabsorptive procedures involved pose a higher risk of deficiencies of vitamins and minerals.^21^ However, studies assessing drug use and their respective dosages after bariatric surgery are still scarce.

## OBJECTIVE

Within this context, this study aimed to assess the impact of bariatric surgery on medication drug use among morbidly obese patients, before and after the procedure, focusing mainly on the number of drugs used, drug classes and posology.

## METHODS

This was a longitudinal study of before-and-after type, carried out between 2008 and 2011 in the endocrinology clinic of the service for assisting morbidly obese individuals at Hospital Nossa Senhora da Conceição (HNSC) in the city of Porto Alegre, which works under the National Health System (Sistema Único de Saúde, SUS). Through this service, four to five operations per month are performed on patients coming from several regions of the state of Rio Grande do Sul. The clinic offers care provided by physicians (endocrinologist, surgeon and psychiatrist), psychologists, nutritionists and nurses.

The patients eligible for surgical treatment are those with BMI greater than 40 kg/m² or greater than 35 kg/m² in association with comorbidities, after failure in applying traditional measures for weight loss. They need to be psychologically capable of following dietary orientation during the postoperative period, as well as presenting absence of endocrine causes of obesity.^22^^,^^23^^,^^24^

The sample size was estimated to be 52 patients, taking into consideration the reduction in drug use for treating hypertension and cardiovascular disease (HT/CVD) that has been reported in the literature.^25^ The reduction in drug use expected after the intervention was 49%. Considering the probability that some patients would be lost from the follow-up, it was decided to increase the sample size by 30%. Thus, after the research project had been approved by the HNSC Research Ethics Committee (report no. 146/08), and after potential participants had signed an informed consent statement authorizing data use, 69 individuals with morbid obesity (convenience sampling), who were waiting for biliopancreatic diversion with duodenal switching (BPD-DS) and Roux-en-Y gastric bypass (RYGBP), were interviewed.

Individuals aged over 18 years with class III obesity (body mass index, BMI, greater than 40 kg/m^2^) and those with BMI greater than 35 kg/m^2^ in association with comorbidities, who were willing to participate in the study, had presented stable obesity for at least five years and had had at least two years of previous inefficient clinical treatment, were included in this study. Patients who did not adhere to preoperative monitoring appointments, had comprehension difficulties (which made them unable to make decisions) or had already undergone another surgical procedure with the aim of losing weight (gastric sleeve) were excluded from the study.

Data were gathered through 40-minute interviews that were conducted with all patients one day before the procedure and again six months after it. A structured questionnaire was used to assess the variables of gender, age, weight, drug use, reasons for undergoing the surgery and comorbidities. To confirm the presence of comorbidities, drug use, weight and height, some data from the patients’ records were used.

Information on drug use at the time of the interviews was initially gathered through the open question “What medications do you take?” For each drug, the name, daily dose, indication and whether its use was through self-medication or prescription were registered. To minimize data loss through forgetfulness, the participants were also asked about their drug use by specifying organs or systems (for example: “Do you take any drug for heart disease?”).

The proportion of patients using each drug was calculated taking into consideration the total number of patients using the drug at each time: preoperatively, n = 69; postoperatively, n = 64. To evaluate dose variability, the sum of the doses of each drug divided by the number of patients using this drug at each time was calculated.

The data were double-input into the Epi Data 2.1a. software. They were analyzed through the Statistical Package for the Social Sciences (SPSS) version 18.0 and the Winpepi software. Comparative and descriptive analyses were performed, expressing frequency, average, standard deviation and P-values. McNemar’s chi-square test was applied to compare the numbers of patients using drugs before and after the procedure. Student’s t test was applied to analyze the dosages. P values ≤ 0.05 were considered significant.

## RESULTS

During the preoperative period, 69 patients who were scheduled to undergo bariatric surgery were monitored. There were no refusals to participate. However, after the procedure, there was a loss of five cases: four patients did not return to the consultations in the hospital and one patient died. The majority of the patients participating in this study were women (91.3%) and married (46.5%). Their mean age was 42.3 (± 10.4); they had a mean of 9 (± 2.3) years of education and had undergone a mean of 4 (± 1.3) years of preoperative monitoring. Their mean BMI before the operation was 51.07 (± 7.8) kg/m², and after the operation it was 35.9 (± 7.2) kg/m^2^ (Table 1).

**Table 1.**
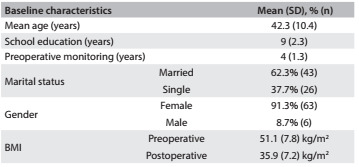
Characteristics of patients undergoing bariatric surgery at Hospital Nossa Senhora da Conceição between 2008 and 2011 (n = 69)

SD = standard deviation; BMI = body mass index.

During the preoperative period, 85.5% of the patients presented one to five comorbidities associated with obesity, with a mean of 2.33 (± 1.47) per patient. The patients between 20 and 25 years of age did not present any comorbidities. On the other hand, comorbidities were present in all patients aged over 49 years. Hypertension, diabetes, high cholesterol level, hypothyroidism, arthrosis/arthritis, asthma, depression and circulatory problems were the main issues reported prior to surgery. After the procedure, significant reductions in hypertension and diabetes were observed. Dyslipidemia, hypothyroidism, arthrosis/arthritis and depression presented reductions, but not significantly (Table 2).

**Table 2.**
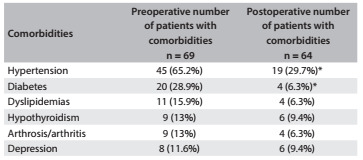
Main comorbidities shown at pre and postoperative assessments

*P ≤ 0.05 (chi-square test).

The surgical technique most often used was gastric bypass (91.4%). The subjects mentioned the following as the main reasons for undergoing the surgery: desire to have better health (42.9%), desire for better quality of life (11.1%), desire for weight loss (11.1%), tiredness (6.3%), difficulty in moving around (6.3%), sore legs (4.8%), backache (3.2%) and prejudice (3.2%).

Among the 69 patients interviewed before the surgery, 84.1% reported using drugs through medical prescription, whereas 73.9% did this through self-medication. At the time of the postoperative assessment, 82.6% and 50.7% took drugs, respectively with and without prescription. Use of 328 drugs was observed before the operation, with an average of 4.8 drugs per patient, whereas after the operation this use reduced to 284 drugs, with an average of 4.4 per patient.

A significant reduction in the use of antihypertensive drugs (49.5%) was observed, except for propranolol. Decreases in the use of antidiabetic (84%), antidepressant (30%), antilipemic (50%), muscle relaxant (33.3%), painkiller (35.3%) and anti-inflammatory (78.5%) drugs were also observed, and these decreases were significant for the following drugs: metformin, fluoxetine, simvastatin, acetylsalicylic acid (ASA), diclofenac and paracetamol. On the other hand, there were significant increases in the use of vitamin supplements and drugs relating to disorders of gastrointestinal tract, such as omeprazole (Table 3).

**Table 3.**
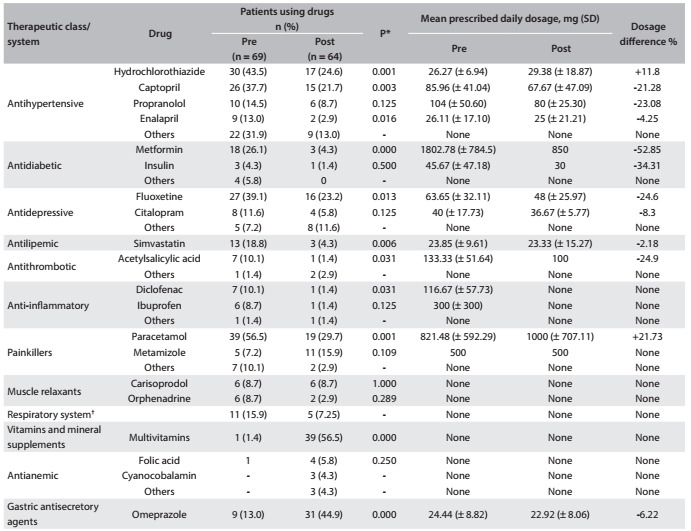
Differences observed in relation to the main therapeutic classes, drugs and mean daily dosages used before and after bariatric surgery

SD = standard deviation; *McNemar’s chi-square test; ^†^oxymetazoline (1 patient), xylometazoline (1), salbutamol (2), fenoterol (1), salmeterol and fluticasone (1), formoterol and budesonide (2), beclometasone (1), dexchlorpheniramine (1) and loratadine (1).

Moreover, reductions in daily dosages were observed for antidiabetic, antilipemic, antidepressant, antithrombotic and most antihypertensive drugs. The differences observed in relation to the dosages were not significant.

## DISCUSSION

Our results showed that use of antidiabetic, antihypertensive, painkiller, antilipemic and antidepressant drugs had decreased by the time of the assessment six months after surgery. However, increases in the numbers of drugs used to treat gastrointestinal disorders, anemia and vitamin deficiency were observed.

Among the patients who kept on using drugs after the surgery, reductions in dosages were observed in most of the cases, mainly in relation to antihypertensive drugs (except for hydrochlorothiazide), antidiabetic drugs and antidepressants; however, these reductions were not significant.

### Antidiabetic drugs

The number of patients using antidiabetic drugs decreased by 84% during the postoperative period. Reductions in mean daily dosages of antidiabetic drugs were also observed. Our results are consistent with those of other authors. Maciejewski et al.^26^ reported that there was a 50% reduction in the use of antidiabetic drugs within one year after bariatric surgery. Additionally, Potteiger et al.^27^ and Narbro et al.^28^ reported that there were significant reductions in the numbers and cost of drugs used to treat diabetes relating to obesity, after surgery. Importantly, the features of the population and the follow-up period need to be taken into account in assessing these proportions, because the impact of surgery on antidiabetic drug use may vary according to these factors.^29^

The great majority of the patients with type 2 diabetes experience more favorable results from clinical examinations, after undergoing bariatric surgery. The mortality rate associated with diabetes has also been significantly reduced.^10^ A more recent study that assessed the long-term effects of bariatric surgery on diabetic patients observed that glucose control and remission from diabetes were possible in 89.2% and 64.7% of the patients, respectively.^30^ Similar findings had already been reported previously.^31^

Furthermore, reduction of diabetes has been found to occur more frequently among patients who underwent gastric bypass surgery that excluded the duodenum from the nutrient pathway and changed the bowel metabolism, thus reducing insulin resistance faster.^32^ Therefore, the significant decrease in antidiabetic drug use observed in our study might be related to the surgical technique used on most subjects (91%).

Recent studies have also suggested that the remission mechanism of this comorbidity in the postoperative period may be categorized into two groups: unconnected with weight loss and connected with it. Although weight loss is an aspect common to all techniques, gastric bypass has shown improvement in diabetes over the short term, regardless of weight loss.^33^^,^^34^ The underlying mechanisms for this are still being studied.^35^^,^^36^,^37^,^38^^,^^39^^,^^40^^,^^41^^,^^42^

Metformin, the most widely used antidiabetic drug, is absorbed slowly and incompletely by the gastrointestinal tract, mainly from the small intestine onwards. Studies suggest that this drug reaches saturation of absorption, since its concentration in the plasma does not increase with administration of ever-higher dosages.^43^

Metformin dosage has to be carefully individualized, based on patients’ tolerance and response. Side effects, especially gastrointestinal effects, are observed in approximately 5 to 50% of the patients and seem to be related to dosage.^43^

Regarding pharmacokinetics, few studies have focused on antidiabetic drug absorption after bariatric surgery. However, Aron-Wisnevsky^44^ observed that metformin bioavailability seems to increase after gastric bypass, thus increasing the risk of toxicity. Therefore, the decrease in dosage observed in our study might be partly related to these findings.

### Antihypertensive drugs

A reduction in the number of patients using antihypertensive drugs during the postoperative period was observed (49.5%) The mean daily dosages became smaller for most drugs, except for hydrochlorothiazide. Increases in dosage may be due to exclusion of other antihypertensive drugs. According to the BAROS system (Bariatric Analysis and Reporting Outcome System), arterial hypertension is resolved after bariatric surgery when patients continue to use diuretics alone.^45^^,^^46^

Regarding the reduction in the number of patients who were using antihypertensive drugs, our findings were similar to those reported by other authors.^26^^,^^27^^,^^28^^,^^47^^,^^48^ Partial or complete improvement was shown within 12 months after undergoing bariatric surgery, especially among the patients who had undergone gastric bypass,^10^^,^^47^ thus reducing the need for antihypertensive drugs.^49^^,^^50^ It seems again that our results were somehow related to the technique used for the majority of our patients, i.e. gastric bypass.

Reductions in plasma catecholamines and renin activity brought about by weight loss is associated with decreased sympathetic activity, and is likely to be a determining factor for controlling hypertension.^51^^,^^52^^,^^53^ Thus, it can be suggested that these factors may have contributed towards decreased antihypertensive drug use after loss of excessive weight due to the bariatric surgery.

### Antilipemic drugs

Approximately 50% of the patients undergoing surgery for weight loss present dyslipidemia, which is a major factor relating to morbidity and mortality rates.^16^^,^^54^ Weight loss significantly improves patients’ lipid profiles. Reductions in triglycerides, total cholesterol and low-density lipoprotein (LDL) levels, and increases in high-density lipoprotein (HDL) occur. Within less than one year, most patients who previously needed lipid-lowering drugs are able to discontinue their use.^16^

Our results confirm the findings from previous studies.^16^^,^^54^ A marked decrease in the number of patients using simvastatin was observed. These results suggest that not only was the surgery effective for weight loss, but also it was an efficient alternative for treating dyslipidemia among these severely obese individuals. Improvement in lipid profile is related to the technique used, and disabsorptive techniques cause more significant changes. However, the mechanisms involved in dyslipidemia reduction following bariatric surgery have not been clarified yet.^10^

Regarding the dosage of antilipemic drugs, there are some reports on atorvastatin in the literature, suggesting that its bioavailability increases after the surgical procedure, thus allowing reduction of the dosage.^44^ Nevertheless, it is not known whether the same occurs with simvastatin. In our study, for patients who continued using simvastatin after surgery, there was no clinically significant reduction in daily dosage use.

### Multivitamins and antianemic drugs

Multivitamin supplementation is recommended during the postoperative period, in order to correct nutritional deficiencies,^55^^,^^56^ especially those relating to vitamins B12, A and D, thiamine, folate and minerals such as iron, zinc and calcium.^57^^,^^58^ Such deficiencies frequent occur after bariatric surgery and relate to decreased food intake and physiological changes produced by the surgery.^59^^,^^60^

Gastric bypass changes how food passes along the gastrointestinal tract and leads to poor nutrient absorption, given that food is exposed to the jejunum earlier than usual, through exclusion of part of the gastric and duodenal surface.^61^ The absorbent surface area and solubility, and consequently drug bioavailability, are affected by this technique.^44^^,^^57^

Our results support previous findings in that they indicated that there was a significant increase in vitamin use that might be related to the surgical technique (91%), as well as decreased food intake after the surgery. Furthermore, an increase in the use of antianemic drugs during the postoperative period was observed. Iron deficiency during this period was very evident.^21^

Gastric antisecretory agents

Unlike our study, in which a significant increase in the use of antisecretory agents was observed, Crémieux^55^ and Fontana and Wohlgemuth^62^ reported reductions in the use of these drugs for up to three years after the surgery. This decrease might be connected with the reduced occurrence of gastroesophageal reflux over time, which might remain stabilized for up to three years. The difference in the results reported by these authors,^55^ in comparison with our study, might be associated with the duration of the postoperative follow-up among the patients and with the standard of service rendered.

Development of stomach ulcers is one of the biggest and most common complications associated with the gastric bypass technique,^63^^,^^64^ and it is reported in 1% to 20% of the patients after surgery.^65^^,^^66^ Stomach ulcers may develop over the short term, possibly associated with technical problems at the intervention site.^67^ They are usually located in the damaged intestinal mucosa, unable to withstand acidity; they may also be located near the anastomosis.^66^ Stapling during the surgery, use of anti-inflammatory drugs or presence of *Helicobacter pylori* (*H. pylori*) during the preoperative period may provoke development of late ulcers. Previous reports in the literature suggest that *H. pylori* damages the mucosal barrier, and that this damage persists into the postoperative period. This induces exacerbation of the ulcer, even if the organism has already been treated.^63^^,^^68^^,^^69^ All these factors may have influenced the increased prescription of antisecretory drugs observed in this study.

### Antidepressants

There are few studies relating to the effect of bariatric surgery on the use of drugs that act on the central nervous system. One of the reasons for this is the screening that is done on patients before they undergo bariatric surgery, given that the presence of moderate or severe psychosis or dementia is one of the exclusion criteria.^55^ Unlike most other studies, ours showed that the use of antidepressants decreased among the patients after the surgical procedure. Use of some classes of antidepressants, especially selective serotonin reuptake inhibitors, has been indicated as an adjunct in treatments for obesity.^43^ This factor may be related to the decrease observed in our study.

Lopes^70^ and Segal et al.^71^ also observed a tendency towards improvement in their patients’ psychological functioning. Nevertheless, it is known that some psychiatric disorders may emerge during this period.

Over the long term, some authors have observed that several psychiatric conditions have been causes of death during the postoperative period, usually through suicide. Depression has been reported to be one of the most frequent late complications (23.4%).^72^^,^^73^^,^^74^^,^^75^

van Hout et al.^76^ pointed out that the psychiatric effects of bariatric surgery might take from 6 to 24 months to emerge. It has been suggested that the levels of anxiety and depression probably will not be significantly different six months after surgery,^77^ and that improvements in depressive conditions might only be observed 12 months after the surgery.^78^

Regarding the daily dosage, decreases were observed in relation to both fluoxetine and citalopram. According to previous reports in the literature, these effects may occur through discontinuation of use of selective serotonin reuptake inhibitors (SSRIs). Therefore, abrupt discontinuation of these drugs should be avoided whenever possible.^43^ The small reductions in antidepressant use and daily dosages observed in our study may have been partially related to this, since complete withdrawal of these drugs may have serious effects.

### Anti-inflammatory drugs and painkillers

The high frequency of use of anti-inflammatory drugs during the preoperative period may be explained by the fact that obesity contributes towards development of inflammatory diseases in the joints. Moreover, anti-inflammatory drugs are efficient possibly because obesity is a proinflammatory condition.^79^

Inflammatory diseases of the joints are more common in obese people probably precisely due to their overweight condition. Therefore, the need for these drugs decreases through surgery,^80^ given that patients have less need for them, for treating pain, fever and inflammation, after they have lost some excess weight.

The use of anti-inflammatory drugs decreased by 78.5%, which corroborated previous findings. Furthermore, there was also a 35.3% reduction in the use of painkillers, and 33.3% regarding muscle relaxants. Among the painkillers, only for dipyrone was there an increase in consumption, which may have been due to the standard prescription issued by the surgical team during the postoperative period. It is possible that this medical prescription may have influenced the choice of analgesic in self-medication situations. It is important to point out that these last two drug classes were the ones most often used in self-medication, thus suggesting that bariatric surgery decreased the need for their use among the patients.

Our results support and reinforce previous findings in the literature regarding the impact of bariatric surgery on the use of some drug classes.^28^^,^^31^

One limitation of our study was the small sample size. However, few studies have assessed dosage reductions among the drugs that continue to be used after bariatric surgery.^81^^,^^82^ Consequently, the data presented in this study provide further details of the benefits of this procedure for patients.

Within this context, there is a need for more comprehensive studies with larger sample sizes and longer follow-up, especially in Brazil, where studies correlating weight loss with drug use, with pharmacokinetic assessments before and after bariatric surgery, are still scarce. Moreover, these studies need to address other factors that may have influenced the increase or decrease in use of some drug classes, such as adherence to treatment, side effects, access to drugs and economic factors, among others.

With the rampant growth of obesity, implementation of prevention policies is also a relevant approach to be considered within public health management, so as not only to prevent obesity but also to prevent its complications due to associated comorbidities, drug use, side effects, reduced quality of life and even societal prejudice.

## CONCLUSION

Based on the data obtained, bariatric surgery was observed to enable decreased need for some of the drug classes used, and also adjustment of the dosages of the drugs that continued to be prescribed. On the other hand, new drug classes were included in the patients’ therapeutic plans, such as vitamins, drugs for gastrointestinal tract disorders and antianemic drugs, as a result of the limitations imposed by the procedure.
